# 

**Published:** 1885-04

**Authors:** 


					﻿v° XXV.
_	& 1855 0’^iA^ vNS
wdTMNffi
JO? (JournaS^^^^
l-/ i by* W.F.WESTMORELAND^fflg <f
Kfe< H.V.M.MILLER.M.D.LL.D.>4a /
LpH	JAMES.A.GRAY. M.D.^kZ
W	<3	>*——J	QJ MSgff
/PUBLISHED BY JAMES.RHARRISON&CgSW?
itPu	Atlanta , ga. JMF
SUBSCRIPTION: $2.50 A YEAR.
				

